# Near-Native Visualization of SARS-CoV-2 Induced Membrane Remodeling and Virion Morphogenesis

**DOI:** 10.3390/v14122786

**Published:** 2022-12-14

**Authors:** Tim Bergner, Fabian Zech, Maximilian Hirschenberger, Steffen Stenger, Konstantin M. J. Sparrer, Frank Kirchhoff, Clarissa Read

**Affiliations:** 1 Central Facility for Electron Microscopy, Ulm University, 89081 Ulm, Germany; 2 Institute of Molecular Virology, Ulm University Medical Center, 89081 Ulm, Germany; 3 Institute for Microbiology and Hygiene, Ulm University Medical Center, 89081 Ulm, Germany; 4 Institute of Virology, Ulm University Medical Center, 89081 Ulm, Germany

**Keywords:** SARS-CoV-2, electron microscopy, transmission electron microscopy, scanning transmission electron microscopy (STEM) tomography, volume EM, high-pressure freezing, freeze substitution, DMV pore, virion assembly, ultrastructural preservation

## Abstract

Infection with the severe acute respiratory syndrome coronavirus-2 (SARS-CoV-2), the causative agent of the COVID-19 pandemic, leads to profound remodeling of cellular membranes, promoting viral replication and virion assembly. A full understanding of this drastic remodeling and the process of virion morphogenesis remains lacking. In this study, we applied room temperature transmission electron microscopy (TEM) and scanning transmission electron microscopy (STEM) tomography to visualize the SARS-CoV-2 replication factory in Vero cells, and present our results in comparison with published cryo-EM studies. We obtained cryo-EM-like clarity of the ultrastructure by employing high-pressure freezing, freeze substitution (HPF-FS) and embedding, allowing room temperature visualization of double-membrane vesicles (DMVs) in a near-native state. In addition, our data illustrate the consecutive stages of virion morphogenesis and reveal that SARS-CoV-2 ribonucleoprotein assembly and membrane curvature occur simultaneously. Finally, we show the tethering of virions to the plasma membrane in 3D, and that accumulations of virus particles lacking spike protein in large vesicles are most likely not a result of defective virion assembly at their membrane. In conclusion, this study puts forward a room-temperature EM technique providing near-native ultrastructural information about SARS-CoV-2 replication, adding to our understanding of the interaction of this pandemic virus with its host cell.

## 1. Introduction

Since the beginning of 2020, severe acute respiratory syndrome coronavirus type 2 (SARS-CoV-2) has infected more than half a billion people and caused over six million deaths worldwide (https://covid19.who.int/ accessed on 5 November 2022). SARS-CoV-2 is primarily transmitted via droplets shed by infected individuals [1].

The spherical or ellipsoidal SARS-CoV-2 virions are composed of a lipid membrane envelope and have a mean diameter between ~85 to 110 nm at their long axis [2,3]. Packaged within the virion is the positive single-stranded RNA (+ssRNA) SARS-CoV-2 genome (~30 kb) that encodes a set of non-structural proteins involved in RNA replication, a variety of accessory proteins playing roles in immune evasion, and four structural proteins [4,5,6,7,8]. The nucleocapsid protein (N) and the viral genomic RNA form a viral ribonucleoprotein (vRNP) complex. The membrane (M) and envelope (E) proteins as well as the spike (S) protein, responsible for the corona-like appearance, are embedded in the host-cell-derived lipid bilayer, forming the virion envelope.

The S protein of SARS-CoV-2 mediates virion attachment to the target cell in an ACE2-dependent manner [9,10,11,12,13]. After entering the cell by S-mediated fusion of the viral and cellular membranes, the viral genome is released into the cytoplasm, starting the replication cycle [10,14]. Similar to other +ssRNA viruses, SARS-CoV-2 replication induces intense remodeling of intracellular membranes [15,16] giving rise to double membrane vesicles (DMVs), presumably originating from the endoplasmic reticulum (ER) [17,18,19], and single membrane vesicles (SMVs) derived from the ER–Golgi intermediate compartment [20,21].

DMVs are the main compartment of viral genomic RNA (vRNA) replication and subgenomic mRNA expression [19,20]. For SARS-CoV-2 and the murine hepatitis virus (MHV), it has recently been discovered using cryo-electron tomography (cryo-ET) and subtomogram averaging that newly synthesized vRNA copies exit the DMVs through a molecular pore complex [22]. These molecular pores span the inner and outer DMV membrane, connecting the interior of the DMV with the cytosol. As virus replication proceeds, the newly formed vRNA localizes to the virus assembly site at the SMV membranes, whereas viral mRNA translocates to the ER and Golgi network for translation and packaging of structural proteins [22]. The newly synthesized M, E and S glycoproteins are transported to SMVs, whereas the N protein associates with the vRNA and clusters at the cytoplasmic face of the SMV membrane [18,20,23,24,25,26]. This is followed by membrane curvature towards the lumen of the SMVs and budding of SARS-CoV-2 virions into the SMV lumen [20,26]. Mature virions exit the cell, presumably via tunnel-like structures that extend from the plasma membrane deep into the cell or by lysosomal exocytosis [26,27].

Important structural insights into the replication cycle and morphogenesis of SARS-CoV-2 have been obtained by electron microscopy (EM). Several groups have imaged structural details of SARS-CoV-2 in the native state by use of cryo-EM [20,22,26], and others have published ultrastructural details obtained by room-temperature EM of conventionally prepared samples (e.g., [4,24,28,29]). Only one group has imaged entire SARS-CoV-2 infected cells by room-temperature 3D EM using conventionally fixed samples [21], and structural investigations of SARS-CoV-2 replication in the (near-)native cellular context remain rare [26]. To study the function of viral and cellular factors in SARS-CoV-2 replication, e.g., with the help of knock-out cell lines, and to aid the development of antiviral compounds interfering with viral replication, a straightforward, reliable and, in the best case, high-throughput EM technique is required that provides optimal preservation of the viral and cellular ultrastructure.

Regardless of the EM technique used, biological aqueous samples require sophisticated preparation procedures to preserve their ultrastructure while ensuring sufficient stability in the vacuum and the electron beam. For conventional room-temperature EM, samples are chemically fixed in aldehydes, dehydrated in a series of organic solvents at increasing concentrations and stained with heavy metals [15,30]. Afterwards, resin-embedded samples are usually ultrathin-sectioned and imaged. The entire preparation procedure can be handled at room temperature, allowing sample preparation with standard EM equipment and processing of many samples in parallel, making the technique suitable for routine high-throughput EM imaging. Furthermore, large samples, e.g., cell monolayers up to entire tissue blocks of several cubic millimeters, can be prepared. Nonetheless, the protocol can result in substantial artefacts such as the alteration of membrane profiles [31]. In contrast, cryo-EM is undoubtedly the least artefact-prone EM technique because the samples are imaged in the frozen hydrated state without introduction of heavy metal compounds. Sample preparation merely consists of sample immobilization by fast freezing, and either the samples are already sufficiently thin to be directly imaged in the cryo-transmission electron microscope (TEM) or need to be thinned with an additional procedure, typically by preparation of TEM lamellae [26,32,33]. However, since frozen hydrated samples are unstable in the electron beam, screening the sample to find a structure of interest is hardly possible and low electron doses must be used for imaging. This results in a low signal to noise ratio, and the averaging of thousands of copies of the same structure is thus necessary to form sufficient contrast and provide the high-resolution data typical for cryo-EM (data with a resolution in the range of a few angstroms). Consequently, cryo-EM is the only appropriate technique to study the molecular organization of virions, macromolecules and small cell organelles, either isolated or within the cell [34,35]. For example, cryo-EM revealed the molecular pore in DMV membranes that could so far not be visualized by room temperature EM approaches [20,22,26]. From this, it was presumed that cryo-EM of native samples is required to allow visualization of the pore. Small samples such as isolated viruses or viral proteins can easily be prepared and imaged by cryo-EM. However, even with the many technical advancements of recent years, larger samples such as cell cultures and tissue samples still need to be prepared using cumbersome procedures requiring specialized equipment and expert human resources [36,37]. Since a routine imaging technique in the field of virus research should allow screening of various samples in parallel and imaging of large volumes, e.g., entire replication factories within the whole cellular context, cryo-EM cannot yet be considered the best method for this purpose [38,39]. One existing method for providing samples with superior ultrastructural preservation compared with conventionally prepared EM samples involves high-pressure freezing (HPF) in combination with freeze-substitution (FS), followed by resin embedding. As the samples are imaged by room-temperature EM, the technique unites the advantages associated with using room-temperature EM of conventionally prepared samples with a near-native structural preservation characteristic for cryo-EM methods. Thus, HPF-FS, often in combination with 3D EM methods, has become the gold standard for routine EM studies of virus–host cell interactions [15,40,41].

In this study, we evaluate the potential of HPF-FS room-temperature EM samples to visualize ultrastructures typical of the SARS-CoV-2 life cycle in cultured cells, including the pore connecting the interior of the DMVs with the cytoplasm, and illustrate SARS-CoV-2 virion assembly, particularly the assembly of the vRNP, in 2D and 3D. Although inherently not providing molecular resolution, our room-temperature EM images of HPF-FS and resin-embedded cells showed the viral and cellular structures with a clarity that was similar to the clarity achieved in published cryo-EM images. Because sample preparation, handling and imaging are less challenging than for cryo-EM, we here offer a potent alternative to cryo-EM and room-temperature EM of conventionally prepared samples for the study of SARS-CoV-2 replication.

## 2. Methods

### 2.1. Cell Culture and Viruses

Vero E6 cells (ATCC) were cultivated in Dulbecco’s Modified Eagle Medium (DMEM, Gibco, Waltham, MA, USA) supplemented with 10% (*v*/*v*) heat-inactivated fetal bovine serum (FBS, Gibco, Waltham, MA, USA), 2 mM L-glutamine (PANBiotech), 100 µg/mL streptomycin (PANBiotech) and 100 U/mL penicillin (PANBiotech) at 37 °C in a 5% CO_2_ atmosphere. The viral SARS-CoV-2 B.1.617.2 (Delta variant) isolate was provided by Florian Schmidt (University of Bonn, Bonn, Germany).

To propagate SARS-CoV-2, Vero E6 cells were inoculated with the SARS-CoV-2 isolate at an MOI of 0.1. Fresh medium was provided six hours post infection and virus stocks were harvested as soon as a strong cytopathic effect became apparent. The virus stocks were centrifuged for 5 min at 1000× *g* to remove cellular debris, aliquoted and stored at −80 °C until further use.

### 2.2. Sample Preparation for Transmission Electron Microscopy and Scanning Transmission Electron Microscopy

Samples for TEM and STEM were prepared following the protocol [40]: Vero E6 cells were cultivated on carbon-coated sapphire discs with a diameter of 3 mm and a thickness of 160 µm (Engineering Office M. Wohlwend GmbH). 24 h after seeding, cells were infected with SARS-CoV-2 with an MOI of 3. Then, 24 h post infection (hpi), the cells were fixed using paraformaldehyde (4%) for 1 h for safety reasons and immediately cryo-immobilized in a Wohlwend HPF compact 01 high-pressure freezer (Engineering Office M. Wohlwend GmbH). Samples were then freeze-substituted in a medium consisting of 0.2% (*v*/*v*) osmium tetroxide and 0.1% uranyl acetate (*w*/*v*) in acetone with 5% (*v*/*v*) water to attain high membrane contrast [42]. Over a period of 17 h, the temperature was raised exponentially from −90 °C to 0 °C with a one-hour incubation step at 0 °C and a further increase to room temperature for one hour. Subsequently, samples were washed thrice with acetone, stepwise embedded in increasing concentrations of Epon 812 (30%, 60% and 100% (Sigma-Aldrich, St. Louis, MO, USA)) in acetone and polymerized at 60 °C for 72 h. The sapphire discs were removed, leaving the cells on the surface of the Epon block.

### 2.3. Transmission Electron Microscopy

For TEM, 70 nm thick sections were cut from the Epon block with a 45° diamond knife (Diatome, Bern, Switzerland) mounted on a Ultracut UCT ultramicrotome (Leica, Wetzlar, Germany) and then collected on freshly glow-discharged 300 mesh copper grids coated with a carbon-reinforced formvar film. Samples were imaged with a JEM-1400 TEM (Jeol, Tokyo, Japan) operating at 120 kV acceleration voltage and images were acquired with a Veleta CCD camera (Olympus, Tokyo, Japan). The diameters of virions were measured in the TEM images using ImageJ (1.53q, [43]).

### 2.4. Scanning Transmission Electron Microscopy Tomography

For STEM tomography, 700 nm thick sections were cut from the Epon block with a 35° diamond knife using an Ultracut UCT ultramicrotome (Leica, Wetzlar, Germany). Sections were collected on freshly glow-discharged copper grids with parallel bars, which had been pre-treated by 10% (*w*/*v*) poly-L-lysine (Sigma Aldrich, St. Louis, MO, USA) in water. After drying on the grids at 37 °C for 5 min, the sections were immersed for a few seconds in a colloidal gold suspension (Aurion, Wageningen, The Netherlands) diluted 1:2 (*v*/*v*) in water so that gold fiducials adhered on both sides of the section, and were finally coated with a 5 nm carbon layer using a BAF 300 (Balzers, Balzers, Liechtenstein) electron beam evaporation device. For STEM data acquisition, a series of 97 STEM bright-field images was recorded at tilt angles from −72° to +72° with a 1.5° increment, using a bright-field detector (EM-24541SIOD, Jeol, Tokyo, Japan) mounted on a JEM-2100F electron microscope (Jeol, Tokyo, Japan) operating at 200 kV acceleration voltage. Tomogram reconstruction was performed as described previously [16] using the IMOD software package vs. 4.7 [44]. For better illustration of sequential budding stages, seven consecutive virtual sections with a voxel size of 1.37 nm each were superimposed. Segmentations of membranes, N and S protein at virion envelopes, and connections between virions and the plasma membrane were carried out manually in Avizo (Thermo Fisher, Watham, MA, USA, version 2020.2). The video sequence was prepared in Fiji ([43], release 2.9.0).

## 3. Results

### 3.1. Overview of a SARS-CoV-2 Infected Cell Shows Prominent Compartmentation

SARS-CoV-2 infection induces profound morphological changes in the host cell, including drastic rearrangement of cellular membranes for efficient virion production [21]. We performed HPF-FS and epoxy resin embedding with Vero E6 cells infected for 24 h, to visualize different stages of SARS-CoV-2 infection and virion morphogenesis and to evaluate the potential of this approach for the study of SARS-CoV-2 interaction with the host cell.

**Figure 1:**
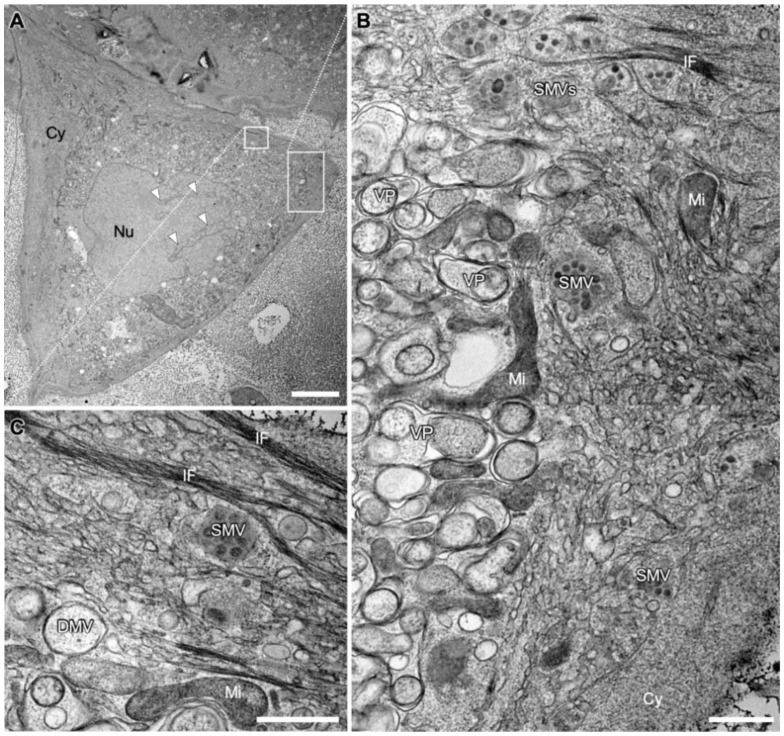
Transmission electron microscopy (TEM) images of a SARS-CoV-2 infected Vero E6 cell at 24 hpi. (**A**) Overview image showing cytopathic alterations, such as the compartmentation of the cytoplasm (Cy). Invagination of the cytoplasm into the nucleus (Nu) is marked with white arrowheads. (**B**) Larger magnification of the indicated area marked in A, illustrating the compartmentation into a perinuclear area on the left and a peripheral area on the right. The perinuclear area is occupied almost exclusively by densely packed double membrane vesicles (DMVs) and numerous vesicle packets (VPs), arising from multiple DMVs fusing at their outer membrane [19,24]. The peripheral area is occupied by cell organelles such as mitochondria (Mi) and endoplasmic reticulum (ER) and single membrane vesicles (SMVs) filled with virions. (**C**) Larger magnification of the area indicated in A with bundles of intermediate filaments (IF), SMV, DMV and a mitochondrion (Mi). Scale bars: (**A**) 10 µm; (**B**,**C**) 500 nm.

We first analyzed a cross section of an entire cell to obtain an impression of the structural impact of SARS-CoV-2 infection on the general architecture of the cell. In the cell shown, the infection presented itself with prominent compartmentation of the cytoplasm into a perinuclear and a peripheral area (Figure 1A). As was shown in higher magnification images of this cell, the perinuclear area was almost exclusively occupied with densely packed DMVs and DMV-related structures (Figure 1B). It surrounded a large portion of the nucleus circumference with a width of several micrometers. Almost no cytosolic space was visible between the DMVs. Thus, and as DMVs are the site of vRNA replication and expression [20], the perinuclear area appeared to be structurally specialized for this early stage of SARS-CoV-2 morphogenesis. Meanwhile, the peripheral area contained numerous SMVs, which are the sites of virion assembly [20]. The spatial arrangement of these two closely apposed compartments might support their functions during the sequential stages of virion morphogenesis and egress: In the early stage, vRNA is replicated in the cell center but virion assembly itself seems to occur preferentially closer to the cell surface, probably to facilitate transport to the cell surface and finally the egress of nascent virions. The peripheral area was also shown to contain cell organelles such as the endoplasmic reticulum (ER), as well as cytoskeletal elements, i.e., intermediate filaments (Figure 1C). In the example shown here, mitochondria were located in the peripheral area or at the interface between the two compartments (Figure 1B), and the cytoplasm formed large invaginations into the nucleus (Figure 1A). However, neither this distinct compartmentation nor the presence of nuclear invaginations were found to be common trends for SARS-CoV-2 infections (Figure S1).

### 3.2. Near-Native Visualization of SARS-CoV-2 Induced DMVs

It has been reported that +ssRNA virus-induced DMV membranes are fragile and that their ultrastructural preservation requires cryo-fixation and optimized FS [18,45,46]. Thus, before being able to analyze SARS-CoV-2 induced DMV membranes in greater detail, we evaluated the quality of DMV membrane preservation after applying our HPF-FS protocols.

**Figure 2:**
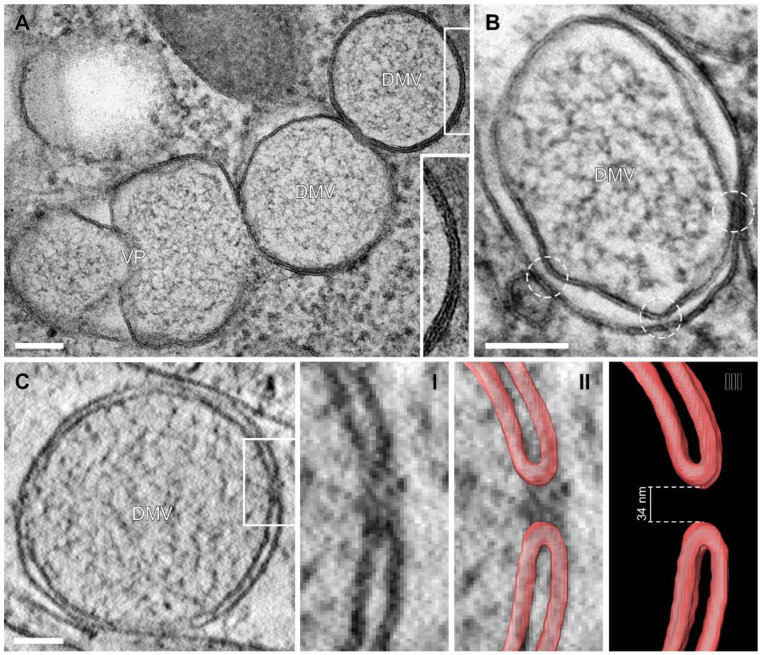
SARS-CoV-2 induced DMVs with inner and outer DMV membranes forming a pore structure. (**A**) TEM images of DMVs and VPs showing tight membrane apposition between inner and outer DMV membrane. An overview of this cell can be found in Figure S1. (**B**) Constrictions of the space between inner and outer DMV membrane (circles), indicating the presence of the molecular pore complex. (**C**) Scanning transmission electron microscopy (STEM) virtual tomography sections of a molecular pore connecting the DMV interior with the cytoplasm. The pore is shown in higher magnification (**I**), together with membrane profiles labelled in red (**II**) or as standalone membrane labels (**III**). Scale bars, 100 nm.

Our samples showed smooth membrane profiles and the two lipid layers of each DMV membrane were resolved as two parallel black lines (hydrophilic head groups of the membrane phospholipids) with a constant distance of ~5 nm, separated by a white line (hydrophobic tails) (Figure 2). Further evidence of near-native sample preservation was the tight membrane apposition between the inner and outer DMV membrane (Figure 2A, insert), usually visible in cryo-EM studies [22] but not in conventionally fixed samples, due to technical limitations [4,45,47]. Our results indicated that pre-fixation with paraformaldehyde followed by HPF then FS for gentle dehydration, with simultaneous contrasting and fixation of the samples by osmium tetroxide and uranyl acetate at subzero temperatures circumvents the relatively harsh conventional dehydration protocol at room temperature [48]. Including 5% water in the FS solution contributed to improved membrane visibility [42]. In our near-native samples, thin filamentous material was present within DMVs, presumably representing vRNA strands [20].

### 3.3. Visualization of the DMV Pore in Room Temperature EM Samples

Results of cryo-electron tomography (cryo-ET) analyses [22] suggest that several molecular pores span both membranes of the DMV, connecting its interior with the cytoplasm, allowing passage of newly synthesized vRNA from the DMV to the ER and SMVs. Since HPF-FS reliably produced visible membrane profiles (Figure 1), we examined whether this method is also suitable for visualizing DMV pores in resin-embedded samples.

DMV cross sections showed two to three constrictions of the space between the inner and outer DMV membrane (Figure 2B, circles). These constrictions have also been observed by cryo-EM in DMVs of Middle East respiratory syndrome-coronavirus (MERS-CoV) infected Huh7 cells after conventional fixation [19] and in SARS-CoV-2 infected cells [20,22,26]. At some of these constriction points, densities connecting the inner and outer membrane were visible. These densities resembled the molecular pore complex at these constriction points visible in cryo-ET virtual sections [22].

We expected that the preparation protocol for our sample might allow direct detection of DMV membrane profiles that form the molecular pore and thus extended our TEM study into the third dimension by conducting STEM tomography. Indeed, in one instance we detected a DMV with a membrane profile resembling the membrane profile surrounding the pore in cryo-ET virtual sections (Figure 2C). The potential molecular pore interrupted both membranes, and the curved ends of the membrane profiles (Figure 2C(II,III), labelled in red) on both sides of the pore were at a distance of 34 nm. This is in a similar range as the diameter of the pore complex observed with cryo-ET (28 nm) [22]. In conclusion, our room-temperature EM approach is useful for the visualization of DMV membranes in a near-native state and provides the good structural clarity crucial for the detection of pore-forming DMV membranes.

### 3.4. SARS-CoV-2 Virion Assembly

After the vRNA reaches the cytoplasm, SARS-CoV-2 virions assemble at the SMV membrane [20]. To gain a better understanding of SMVs and this process, we imaged the site of virion assembly using TEM and in 3D using STEM tomography.

**Figure 3:**
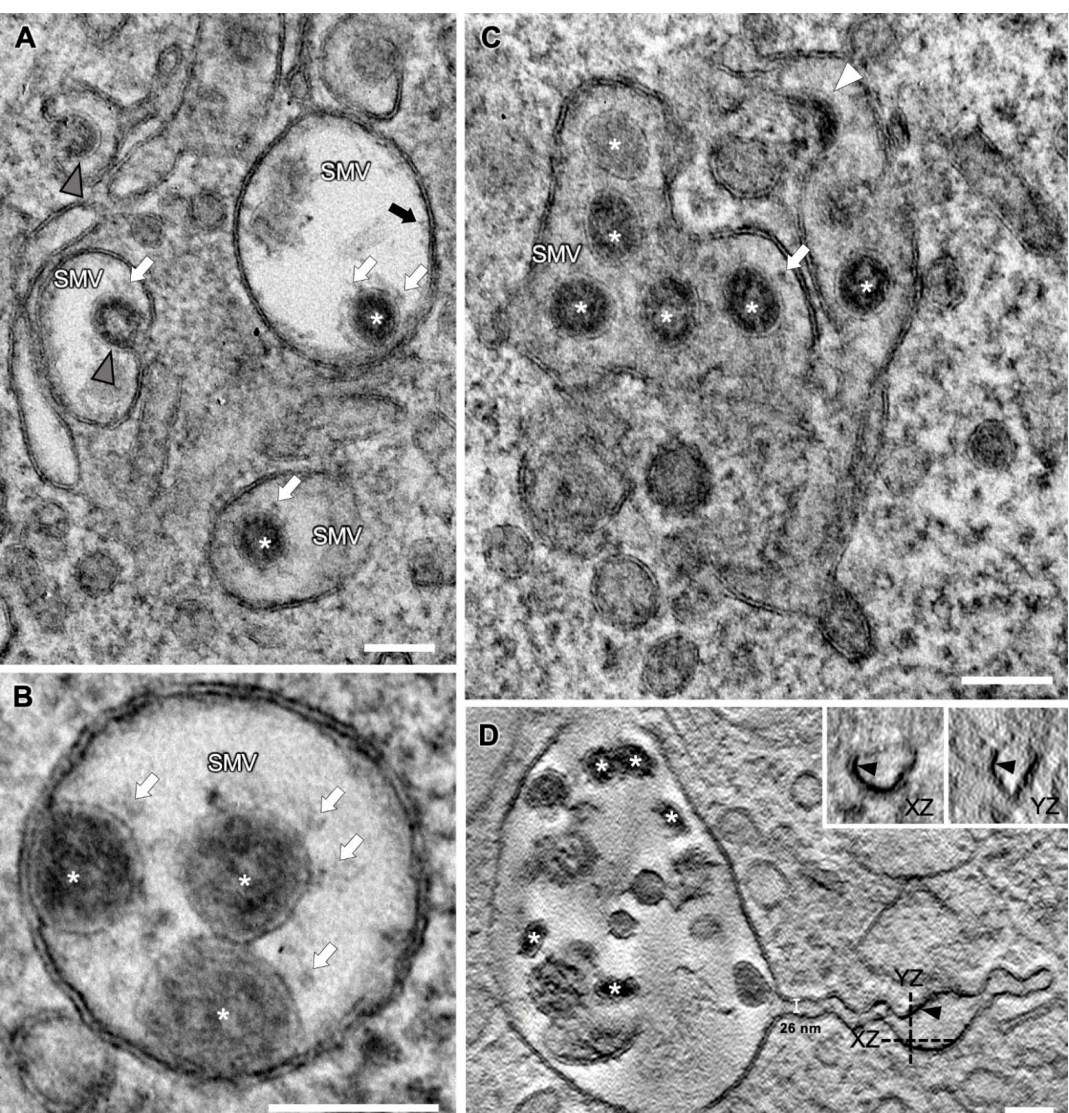
Different appearances of SARS-CoV-2 virion-containing SMVs at 24 hpi. (**A**–**C**) Various SMVs with beginning (white arrowhead) and advanced (grey arrowheads) virion budding stages, and nascent virions (white asterisks). Note the viral S proteins on the virion envelopes (white arrows) and the protein densities on the inner leaflet of the SMV membrane (black arrow). (**D**) Virtual section through a STEM tomogram of an SMV with a tubular extension containing numerous virions (white asterisks). Dashed lines indicate the position and direction of the virtual XZ and YZ sections shown in the insets. Black arrowheads mark a fine seam of electron-dense material at the inner membrane leaflet. Scale bars, 100 nm.

**Figure 4:**
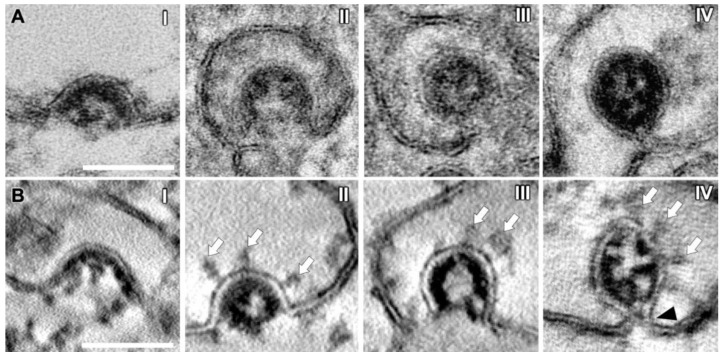
Consecutive stages of SARS-CoV-2 virion assembly. (**A**) TEM images. (**B**) Virtual sections (thickness ~10 nm) generated by STEM tomography. (**I**) N-packaged viral genome clusters at the cytoplasmic membrane face, accompanied by membrane curvature. (**II**) With increasing membrane curvature, the budding event is driven forward, until the membrane profile appears as an Omega shape (**III**). (**IV**) Virions shortly before membrane scission, still connected to the SMV membrane (black arrowhead). Note the improved visibility of the viral ribonucleoprotein (vRNP) lining the inside of the forming virion, the prominent S proteins (white arrows) and the optimal visibility of membrane profiles in the STEM virtual sections. Scale bars, 100 nm.

**Figure 5:**
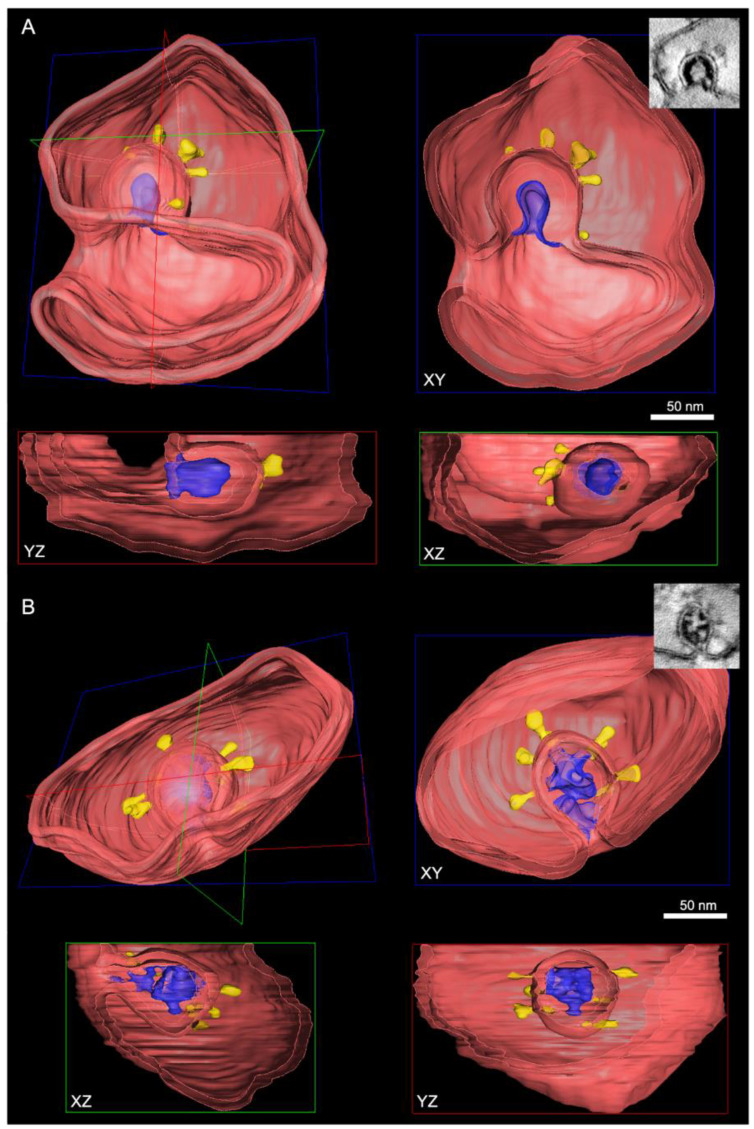
SARS-CoV-2 virion assembly in 3D. The cross-sectioned SMVs are shown in the respective XY, YZ and XZ perspectives. (**A**) Membrane profile at the “Omega stage” of virion assembly (from Figure 4(BIII)). The virion shown here assembles at an invagination of the SMV membrane. (**B**) Virion that is still in contact with the cytoplasm by a narrow bud neck (from Figure 4(BIV)). Red: SMV membrane; blue: nucleocapsid; yellow: S protein associated with the forming virion envelope.

Virion-filled SMVs appeared to be spherical (Figure 3A,B) or tubular (Figure 3C) and exhibited various shapes and sizes (Figure 3, Figure 4 and Figure 5). Multiple budding events at one SMV led to the formation of SMVs that contained multiple virions (Figure 3). Irregularly distributed protein densities, potentially also representing viral S protein, were anchored in SMV membranes, protruding into the SMVs’ luminal space (Figure 3A, black arrow). In STEM tomograms of 700 nm sections, spherical SMVs with thin, tubular extensions could be seen (Figure 3D). The minimum diameter of these tubules was approximately 26 nm. These tubular extensions showed a fine seam of electron-dense material at the inner membrane leaflet, most likely representing membrane-associated protein. Contrast and visibility of the S protein were improved in ~10 nm STEM virtual sections (Figure 3A–C and Figure 4B, white arrows) compared with TEM images, so that the S protein trimer appeared as a thin stem with a wider tip, similar to the visualization revealed by cryo-EM [49]. TEM and STEM images showed that the S protein accumulated at the portions of the SMV membrane which were engaged in virion assembly (curved membrane lined with vRNP, Figure 4). This agrees with the model derived from cryo-EM imaging data, stating that S protein clusters at the assembly site in the presence of vRNA and N protein [26]. STEM imaging allowed 3D imaging of large SMVs including virion assembly sites, and revealed that virions also assembled at invaginations of the SMV membrane (Figure 5A).

### 3.5. SMV Membrane Curving and Nucleocapsid Assembly Occur Gradually and Simultaneously

To illustrate the virion assembly process, we selected four distinct budding intermediates visible in virtual sections (thickness ~10 nm) acquired by STEM tomography, put them into a putative temporal order and displayed them alongside similar intermediates acquired by TEM (Figure 4). Virion assembly was initiated by accumulation of electron-dense material, the vRNP, at the cytoplasmic face of the SMV membrane and curvature of the membrane portion lined with vRNP into the SMV lumen (Figure 4(AI,BI)). At this stage of the assembly process, the vRNP appeared in the STEM images as a string of beads, indicating that the vRNP had already formed before it reached the SMV membrane [50], but only bound to the membrane upon initiation of membrane curvature it started assembling into the nucleocapsid. Assembly of the vRNP/nucleocapsid apparently occurred not suddenly but gradually, accompanied by further curvature of the SMV membrane (Figure 4(AII,BII)), until the membrane formed an Omega profile (Figure 4(AIII,BIII)) and the bud neck became visible (Figure 4(AIV,BIV), black arrowhead). After a membrane fission and fusion event at the constricting bud neck, the nascent virion was released into the SMV lumen (Figure 3, asterisks). Finally, nascent virions in SMVs appeared in circular to oval shapes (Figure 3, asterisks), with the S protein protruding from their envelopes (Figure 3, white arrows). Viral S protein was more dispersed on the viral envelopes of nascent virions further away from the membrane, compared with budding virions (Figure 3 and Figure 4). This agrees with cryo-ET data showing that the S protein is redistributed during the budding process [20]. Within SMVs, nascent SARS-CoV-2 virions then reached the plasma membrane, obviously facilitated by the close vicinity of the assembly sites and the cell surface (Figure 1). Virions were then released into the extracellular space via lysosomal exocytosis [20,51,52].

The vRNP inside virions was visible as individual black dots, similar as in cryo-EM data [20,22,26], covering the entire inner leaflet of the forming virion envelope at all stages of the assembly process (Figure 4 and Figure 5). This showed that membrane curvature and nucleocapsid assembly proceeded simultaneously. S protein-studded membrane portions showed curvature only when this vRNP layer was present (e.g., Figure 4). We also observed that in the process of budding, SARS-CoV-2 virions already exhibited a cross section that was oval rather than circular, but never angular (Figure 3, Figure 4 and Figure 5).

Taken together, our results show that vRNP is already formed when reaching the SMV membrane, that membrane curving and nucleocapsid assembly occur gradually and simultaneously, and that the variations in SARS-CoV-2 virions’ shape and size originate from the budding process.

### 3.6. SARS-CoV-2 Virion Tethering

**Figure 6:**
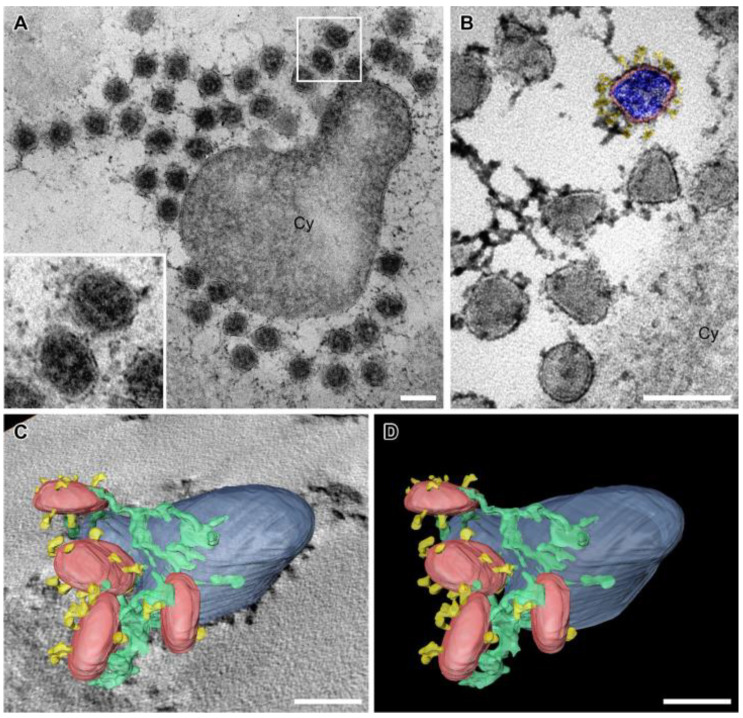
Accumulation of extracellular SARS-CoV-2 virions with prominent filamentous material between individual virions and between virions and the cell (Cy). (**A**) Virions (higher magnification in the insert) around a cross-sectioned cellular protrusion. (**B**) Virions at the cell surface. One virion is colored. Red: viral envelope; blue: nucleocapsid; yellow: S protein. (**C**,**D**) 3D segmentation of STEM tomography data showing tethered virions (red) with S proteins (yellow) connected by filamentous material (green) at the plasma membrane (blue). Full dataset shown in Supplementary Video S1. Scale bars, 100 nm.

After release from the cell, circular to oval extracellular virions with prominent vRNP accumulated at the cell surface (Figure 6A). Furthermore, virions with angular cross sections were present (Figure 6B), possibly representing virions from initial infection. However, we cannot exclude that these angular cross sections were an artefact of dehydration.

Extracellular accumulations of SARS-CoV-2 [26,53], SARS-CoV-1 virions [54] and other enveloped viruses, e.g., human immunodeficiency virus-1 (HIV-1) [55,56], have previously been imaged by TEM. However, to our knowledge, ultrastructural evidence for SARS-CoV-2 virion tethering has been lacking, although it has been reported that SARS-CoV-2 release is inhibited by tetherin, which is the molecule that tethers virions to the cell surface [57]. Our TEM and STEM approach directly visualized SARS-CoV-2 virion tethering as filamentous material connecting individual virions with each other and with the plasma membrane (Figure 6, Supplementary Video S1).

### 3.7. Cellular Degradation of SARS-CoV-2 Virions

**Figure 7:**
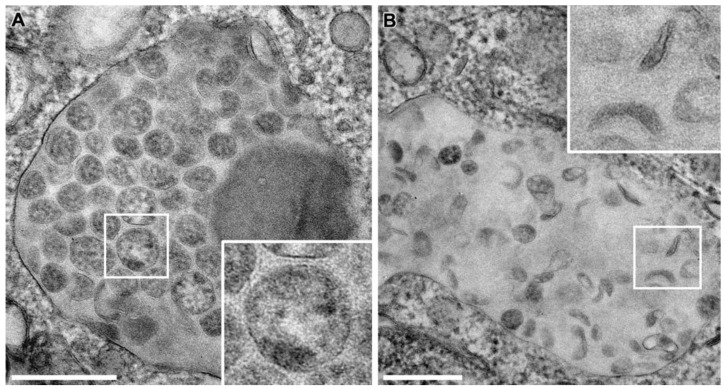
TEM images of large intracellular compartments containing SARS-CoV-2 virions lacking S protein in Vero E6 cells at 24 hpi. Virions within these compartments were (**A**) circular/oval or (**B**) spindle and sickle-shaped. None of the virions exhibited S protein on their surface. Scale bars, 250 nm.

Previously, intracellular compartments containing numerous S protein-lacking particles have been reported [4,20,26,58]. These particles were proposed to be incorrectly assembled or degraded virions. To understand the origin of these particles, we analyze the intracellular compartments by TEM (Figure 7). Notably, the particles they contained were not only devoid of S protein but unambiguously exhibited vRNP (Figure 7A). However, vRNP was less pronounced than in budding or nascent virions (Figure 3 and Figure 4). In addition, the lumen of the compartments was not so electron translucent as the lumen of SMVs (Figure 3 and Figure 4) but was filled with a diffuse density and structures that appeared degraded (Figure 7A), arguing for its lysosomal origin [59]. In line with this, the contained particles exhibited different shapes from oval or circular to sickle- or spindle-shaped (Figure 7B), suggestive of degradation. Most importantly, we did not observe any budding events at the corresponding membranes (Figure 7). These observations suggest that the S protein-lacking particles in intracellular compartments might not arise from a defective assembly process at the membranes of these compartments but are possibly the result of degradation taking place after virion assembly.

## 4. Discussion

In this study, we achieved near-native ultrastructural insights into the different stages of SARS-CoV-2 morphogenesis in the cellular context. We illustrated DMVs with vRNA and followed the process of virion morphogenesis from DMVs via virion assembly at SMV membranes to tethering of extracellular virions to the plasma membrane, in 2D by TEM and 3D by STEM tomography. We furthermore suggest that the deformed virions in the large cytoplasmic compartments are not the result of defective virion assembly within these compartments but might rather be the result of cellular degradation.

These findings are based on the excellent ultrastructural clarity provided by sample preparation using HPF-FS. The near-native structural preservation of these room-temperature samples was particularly evident when comparing the appearances of structural details—such as the pore interrupting the two membranes of the DMVs (Figure 2C), the tight apposition of the two DMV membranes (Figure 2A), the smooth membrane profiles of SMVs and the clear visibility of S protein and vRNP complex during virion assembly (Figure 4)—with their published appearance in cryo-ET virtual sections [20,22,26]. However, extracellular virions imaged by cryo-EM never appear with angular cross sections as they often did in this study (Figure 6). This might be an artefact of the dehydration process during FS.

This HPF-FS approach allowed us first to analyzing the general layout of an infected cell and its compartmentation into a perinuclear and peripheral area (Figure 1), and then to focus on well-preserved details such as DMVs, SMVs or extracellular virions (Figure 2, Figure 3, Figure 4, Figure 5, Figure 6 and Figure 7). This was possible since samples of up to 200 µm, including entire cells or tissue samples [60], can be well frozen by HPF without the formation of ultrastructure-damaging ice crystals [61]. HPF samples are thus 10-fold thicker than plunge frozen samples, which remains the easiest and most widespread technique for cryo-EM studies but is only optimal for small samples, such as isolated virions or thin parts of cells [35]. The stable room-temperature samples also allowed imaging of the same cell several times without causing damage to the structures. During the FS process, the temperature of the samples is slowly increased from -90 °C to room temperature whilst the samples are gently dehydrated, fixed and stained with heavy metals, and finally embedded in resin [62]. The room-temperature samples suffered less sample shrinkage, reduced osmotic effects, and fewer alterations in membrane profiles compared with conventional EM sample preparation [31,63]. The resin blocks and the sections cut from them remain stable without suffering significant quality reduction over time. Thus, they can be revisited multiple times, facilitating overview images with subsequent detailed analysis as shown above, or enabling quantitative approaches, or can be easily transferred to other EM laboratories which do not have HPF-FS equipment to their disposal. Another advantage of the prepared samples is their versatility, meaning that they can be further processed not only for TEM imaging but also for imaging by 3D EM techniques covering different ranges of resolution and volume, e.g., STEM tomography as performed in this study, or block-face scanning electron microscopy (SEM) and focused ion beam-SEM tomography [15,40,41]. Thus, a comprehensive toolbox for the study of virus–host cell interactions is available with HPF-FS samples.

Naturally, cryo-EM provides the best image quality so that subtomogram averaging or single particle analysis provides structural data with molecular resolution, as demonstrated by the discovery of the molecular pore complex spanning the DMV membranes [22]. In contrast, HPF-FS allowed the localization of the pore in DMV membranes, however, the protein densities contributing to the pore complex itself were most probably masked by gentle but still artefact-forming sample preparation. Since cryo-EM also requires high-end equipment and highly skilled staff for sample preparation, imaging and data analysis, the approach used here is a powerful alternative when a large number of samples requires preparation and imaging in a given time. For a single experiment, up to 24 sapphire discs or more can easily be prepared by HPF and FS at once. Each sapphire disc results in two to three final resin blocks. From one resin block, several ultrathin sections from different heights in the cell can be generated. Each TEM ultrathin section usually contains several tens of cells, meaning that acquisition of images from around 100 cells from one sapphire disc should be possible. In cryo-TEM lamellae preparation, every single cell/cell’s subvolume needs to be prepared in an individual milling process and areas next to the prepared lamella are lost.

The benefit of applying STEM tomography lies in providing additional 3D information and coverage of larger volumes than TEM sections (<100 nm thick) or even TEM tomography sections (<300 nm thick), and a resolution superior to TEM images of ultrathin sections [47,64]. We used these advantages to show several layers of extracellular virions contained within a single tomogram (Figure 6C,D). The improved STEM resolution became evident when the vRNP inside virions could be perceived as individual black dots (Figure 4B), similar to their appearance in cryo-EM data [20,22,26]. The vRNP covered the entire inner leaflet of the formational virion envelope at all stages of the assembly process (Figure 4 and Figure 5), showing that membrane curvature and nucleocapsid assembly proceeded simultaneously. This is in line with findings from other coronaviruses, that the M protein forms a lattice to recruit E, S, N protein and vRNA, and that the presence of the E protein initiates membrane curvature [65]. In our images, this lattice appeared as vRNP layer at the cytoplasmic site of the curving membranes (Figure 4). The S protein-studded membrane portions showed curvature only when this vRNP layer was present (e.g., Figure 4), confirming that the S protein is not sufficient to initiate membrane curvature [20,65,66]. EM provides static images, so we were unable to conclude in which time frame nucleocapsid assembly and envelope formation occurred. Nevertheless, the regular appearance of budding intermediates as well as nascent virions (Figure 3 and Figure 4) at least indicates that virion formation occurs not suddenly but gradually. The observation of budding virions with cross sections that were oval rather than circular, but never angular

(Figure 3, Figure 4 and Figure 5), showed that the cause of variation in virion shapes from circular to oval (Figure 6) must lie in the budding process, possibly resulting from the shape given to the membrane by the clustering vRNP. Various virion shapes during budding have also been observed in native cryo-EM samples [20], suggesting that they do not represent preparation artefacts.

Taken together, our study demonstrates that HPF-FS is a valuable methodological alternative to EM analyses of frozen hydrated samples in cases where a high-throughput technique is required and molecular resolution is not essential. HPF-FS reliably generates high-quality samples for imaging, and most importantly is better accessible for most virologists than high-end cryo-EM techniques. By this method it is possible to study virus–host cell interactions in general and specific ultrastructural aspects of these interactions, e.g., the function of viral or cellular gene products in virion morphogenesis or the effect of drug candidates on the virus. Especially in combination with 2D and 3D EM techniques, HPF-FS is a powerful technique to provide fundamental insights into the replication of SARS-CoV-2 and other viruses.

## Data Availability

No further data available.

## Supplementary Materials

The following supporting information can be downloaded at: https://www.mdpi.com/article/10.3390/v14122786/s1, Video S1: SARS-CoV-2 virion tethering. Segmentation of SARS-CoV-2 virions tethered to the plasma membrane. Red: Virion envelope; yellow: S protein; blue: plasma membrane; green: connections between virions and the plasma membrane. Figure S1: TEM images of a second SARS-CoV-2 infected Vero E6 cell at 24 hpi. (A) Overview image showing an entire cell and the area of the cell magnified in Figure S1B. (B) Larger magnification of the indicated area marked in (A), illustrating DMVs and SMVs. (C) Larger magnification of the area indicated in (B) with SMVs, DMVs and mitochondria (Mi). The box marks from the origin of the DMVs in Figure 2A. Scale bars: (A) 20 µm; (B,C) 5 µm.
